# Valuation of the EQ-5D-5L with composite time trade-off for the German population – an exploratory study

**DOI:** 10.1186/s12955-017-0617-9

**Published:** 2017-02-20

**Authors:** Kristina Ludwig, J.-Matthias Graf von der Schulenburg, Wolfgang Greiner

**Affiliations:** 1 Health Economics and Health Care Management, Bielefeld University, P.O. Box 10 01 31, 33501 Bielefeld, Germany; 2 Center for Health Economics Research Hannover (CHERH), Leibniz Universität Hannover, Otto-Brenner-Straße 1, 30159 Hannover, Germany

**Keywords:** Quality of life, EQ-5D-5L, Valuation, Time trade-off, Discrete choice experiment

## Abstract

**Background:**

The EuroQol Group has extended the severity levels of the EQ-5D from three to five (EQ-5D-5L). There are valuation studies worldwide planned in order to convert the EQ-5D-5L health states into a single preference-based summary score based on country-specific value sets of social health status preference valuations. The EuroQol Group developed an internationally standardised EQ-5D-5L valuation protocol. Based on the experiences of the first wave of valuation studies applying the protocol, a number of modifications to the implementation of composite time trade-off (cTTO) were proposed and tested in an exploratory study in Germany.

**Methods:**

The aim of the study is to test the improved EQ-5D-5L valuation protocol 1.1 and the implementation of three modifications: (1) introduction of ranking task, (2) separating time trade-off (TTO) tasks for health states “Better Than Dead“(BTD)/“Worse Than Dead” (WTD), (3) allow for removal of problematic valuations from the cTTO data (the feedback module). Data were collected in computer assisted personal interviews with 200 members of the German general public.

**Results:**

In comparison to the first wave of valuation studies a higher data quality can be observed in both study arms: increasing number of WTD valuations, reduced inconsistencies for health state 55555 as well as higher values for mild health states. Comparing both study arms, mean observed cTTO value for severity 6 is higher in the test arm. The proportion of inconsistent cTTO responses is lower in the test arm than in the control arm and is further reduced by the feedback module. The ranking task prolongs the interview without the desired effect.

**Conclusions:**

Both study arms yielded higher data quality in comparison to the first wave of EQ-5D-5L valuation studies. The valuation protocol combined with an intensive interviewer training and close data monitoring showed a high feasibility and acceptability to the respondents of the general population as well as the interviewers in Germany. Based on the results of this study and other countries, the separation of TTO tasks for health states BTD/WTD and the feedback module will be implemented in the valuation study for the EQ-5D-5L for Germany.

## Background

The EQ-5D 3-level version (EQ-5D-3L) is a multidimensional instrument for the measurement of health-related quality of life (HrQoL) developed and provided by the EuroQol Group. The questionnaire allows collecting descriptive data on individuals’ HrQoL profile on five dimensions (mobility, self-care, usual activities, pain/discomfort, anxiety/depression). Furthermore, it includes a self-rating of health status on a visual analogue scale (EQ VAS) ranging from 0 to 100 [1]. By now the EQ-5D-3L belongs to the most commonly applied generic HrQoL instruments in Germany [2]. There is an increasing demand for the EQ-5D-3L and the corresponding value set in Germany (not least because Germany is a country with a great market volume for pharmaceuticals). Moreover, the German version of the EQ-5D-3L and its value set is applied in studies in other German-speaking countries like Austria and Switzerland.

To increase the sensitivity and to reduce ceiling effects of the existing EQ-5D-3L, the EuroQol Group raised the number of answering levels from three to five (no, slight, moderate, severe and extreme problems/unable to) in a new version of the questionnaire, the so-called EQ-5D-5L (5-level version), which allows to describe 3,125 (=5^5^) health states [3, 4].

In contrast to the EQ-5D-3L, there is only a crosswalk value set available to convert each EQ-5D-5L health state into a single preference-based summary score as there is currently no set of social health status preference valuations available for the German context [5, 6]. The current value set for the German version of the EQ-5D has been generated more than ten years ago [7] and there is also a demand for a new valuation, because the questionnaire has been changed from a 3-level to a 5-level version. In order to use the extended instrument in cost-utility analysis it is essential to generate such a value set for the German context. Combining these arguments, there is an urgent need for an updated value set for the 5-level EQ-5D version in Germany.

The EuroQol Group has developed an internationally standardised protocol with an accompanying computer-based valuation software (the EuroQol – Valuation Technology version 1.0, EQ-VT 1.0) for the valuation of the EQ-5D-5L [3, 8]. In the first wave of EQ-5D-5L valuation studies applying the protocol, major data issues were observed, particularly with respect to the composite time trade-off (cTTO) (see detailed description of the data issues elsewhere [9]). Firstly, quality issues concerning the cTTO distribution suggest that the discriminative capability of the valuation task in the EQ-VT is too low to adequately deal with the increased severity of the EQ-5D-5L descriptive system: few observations between −0.5 and 0, spikes (i.e. clustering of valuations at −1, −0.5, 0, 0.5, 1) and lower than expected values for mild health states (i.e. a big gap to 1). Extending the descriptive system from three to five levels aimed at increasing the discriminative capacity and the sensitivity to change in comparison to the EQ-5D-3L as well as reducing the ceiling effects, especially for small changes in health and for patients with mild health problems [4]. Therefore, the valuation task of the extended instrument must also be sufficiently responsive to these small changes, indicating that the respondents can distinguish between these health states [9]. Moreover, due to the labelling of level 2 as “slight problems” in the EQ-5D-5L a small gap from the perfect health to the next best health state is expected [10]. Secondly, there was a high number of inconsistencies overall and with regard to the worst possible health state 55555 with the highest level of health problems on each dimension (i.e. valuing less severe health states lower than the value for 55555). This quality issue might undermine the face validity of the results as well as impair the data modelling. Thirdly, another quality issue was related to the low number of health state valuations “Worse Than Dead” (WTD) which might be an artefact of the valuation methodology in the EQ-VT [9–11]. The resulting narrow value range due to low values for mild health states and high values for severe health states was not expected. The selective scale use in the EQ-VT and the possible causes need to be further investigated. Shah et al. assumed that those data quality issues could relate to the way in which the cTTO tasks were implemented in the EQ-VT 1.0 [9]. Moreover, post-hoc analyses of the data collected in the EQ-5D-5L valuation studies in Spain and in the Netherlands indicated the presence of interviewer effects with respect to both compliance to the EQ-5D-5L valuation protocol and the cTTO values elicited [11–14].

Based on the experiences of the first five EQ-5D-5L valuation studies, three modifications were immediately added to the software and led to the EQ-VT version 1.1: (1) the use of routine quality control (QC) checks, (2) the introduction of three practise cTTO tasks (a mild health state, a severe health state and a health state which is hard to imagine), and (3) the inclusion of a prompt each time the respondent completes a cTTO task. Moreover, a number of other solutions were worked out to address the described quality issues with the EQ-VT 1.0 and to further develop the valuation methodology for the EQ-5D-5L. Those solutions were tested in a multi-country research programme [9].

Besides explorative studies in Spain, the Netherlands, the UK and other countries, an experimental study was undertaken in Germany as part of the EQ-VT methodology research programme. The objective of this study was to test the improved EQ-5D-5L valuation protocol (EQ-VT version 1.1) and the effect of adding three further modifications to the current EQ-VT version 1.1 to improve the software for further EQ-5D-5L valuation studies.

## Methods

**Fig. 1:**
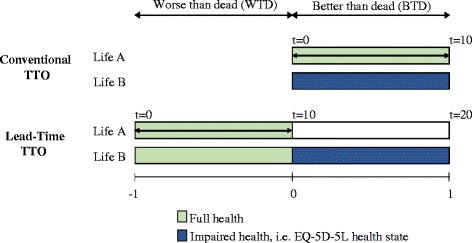
The composite time trade-off

This study used the latest development in valuation methodology in accordance with the EuroQol Group’s protocol and guidelines for the implementation of the protocol for the EQ-5D-5L valuation studies [8]. A combination of two valuation elicitation techniques, including a time trade-off (TTO) approach and a discrete choice experiment (DCE), were applied in this study. Both are choice-based techniques where the respondent has to choose between different alternatives. In case of the TTO, individuals are asked to indicate the amount of remaining life years in full health, after a series of choice-based iteration steps, at which the respondent is indifferent between a longer period of impaired health (i.e. an EQ-5D-5L health state) and a shorter life span in a state of full health [15]. Thereby, a cTTO approach is used which involves beginning with the ‘conventional’ TTO for all EQ-5D-5L health states, and shifting to a ‘lead time’-TTO (LT-TTO) where the participants’ responses indicate that they consider the health state to be worse than being dead (see Fig. 1) [3, 12, 16, 17]. Applying the DCE, respondents have to make a discrete choice between two alternatives (e.g. two EQ-5D-5L health states) according to their preferences [18, 19]. The TTO method belongs to the most widely used and accepted preference elicitation techniques. The DCE has attracted a lot of attention in the area of health evaluation, as its usage offers several advantages (e.g. the ease of comprehension and administration, and the greater reliability due to a reduced measurement error) [18, 20].

The study was conducted according to the standardised protocol (e.g. design, selection of health states and instructions for the interviewers) and used a standardised software platform for the valuation studies, the EQ-VT version 1.1. In this study the implementation of the following three modifications to the current EQ-VT version 1.1 was analysed:

1. Ranking task: introduction of a ranking task prior to the cTTO valuation,
2. “Better Than Dead” (BTD)/WTD split: separating the TTO tasks for health states BTD and WTD,
3. Feedback module: presenting respondents with the rank ordering implied by their cTTO responses and allowing for removal of problematic valuations from the data.

Table 1 summarises the three modifications and the hypotheses being tested in this study. Those three modifications were also tested in other experimental studies of the EuroQol Group: the ranking task (Spain [21], Japan), the BTD/WTD split (Hong Kong, the Netherlands [22]) and the feedback module (e.g. Spain [21], the Netherlands [22]) [9].

**Table 1:** Overview of modifications and hypotheses tested in this study

Modification	Hypotheses tested
Ranking task	Introducing the ranking task prior to the cTTO valuation will reduce the inconsistencies and improves the overall data quality (i.e. more WTD responses).
BTD/WTD split	Separating the BTD and WTD cTTO tasks will reduce the inconsistencies of the valuations and improve the overall data quality (i.e. more WTD responses, higher values for mild heath state and fewer spikes).
Feedback module	Presenting respondents with the rank ordering implied by their cTTO valuations will reduce the inconsistencies as they will identify and flag problematic valuations for removal from the data.

### Methods of eliciting preferences and valuation interview

The EQ-5D-5L descriptive system consists of five dimensions of health with each dimension distinguishing five levels of severity ranging from no problems (1) to extreme problems/unable to (5), which allows to describe 3,125 health states. In this study a sub-set of the 3,125 possible EQ-5D-5L health states was valued. The selection of health states was based on the experimental design which was developed as part of the EQ-5D-5L valuation protocol [8]. For the cTTO tasks an efficient experimental design was generated based on a set of 80 EQ-5D-5L health states, divided into ten blocks of eight health states. The worst possible health state 55555 and one of the five very mild EQ-5D-5L health states were then added to each block, resulting in a total of ten health states per block, balanced in terms of severity. The efficient design developed for the DCE task consists of 196 pairs of EQ-5D-5L health states divided into 28 blocks of seven pairs. None of the pairs included an EQ-5D-5L health state that logically dominates the other. Each respondent was randomly assigned to one of the ten blocks of cTTO tasks and to one of the 28 blocks of DCE tasks. Health states within the blocks were randomly presented to the respondents. The control arm followed the existing protocol for the EQ-5D-5L valuation studies (EQ-VT version 1.1). Each interview in the control arm consisted of the following six parts:

1. Welcome and purpose of the study,
2. Self-reported health using the EQ-5D-5L and background questions regarding age, gender and experience of serious illness,
3. Valuation tasks applying the cTTO (wheelchair example, three practise cTTO states, ten cTTO tasks, three debriefing questions regarding cTTO tasks),
4. Valuation tasks applying the DCE (seven DCE tasks, three debriefing questions regarding the DCE tasks),
5. Opportunity to leave feedback in a comment box,
6. Further background questions specific to the German valuation study (e.g. education, employment status).

### Modification 1: the ranking task

In the experimental test arm, the *ranking task* was introduced before the cTTO task. The ten EQ-5D-5L health states to be valued in the later cTTO task and the state “death” were ranked by the respondents using physical cards as a warm-up exercise. The rank ordering of the respondents was entered into the EQ-VT by the interviewer and determined the order in which the health states will be presented in the cTTO tasks (the ranking and sorting approach): (1) top health state, (2) bottom health state, (3) health state ranked midway between top and bottom health states, (4) health state ranked midway between top and middle health states, (5) health state ranked midway between middle and bottom health states and (6) the remaining health states one after another [21]. Moreover, the ranked health states remained visible on the table and the interviewer always referred to the card representing the health state in the EQ-VT during the cTTO tasks.

### Modification 2: the BTD/WTD split

Before the respondents answer the practise states and the ten cTTO tasks, the interviewer uses the example health state “being in a wheelchair” to explain the cTTO tasks by illustrating all elements and the iterative sequence of the task. The wheelchair example and the practise states serve as warm-up tasks that are usually conducted to prepare the respondent for the formal valuation tasks using the cTTO [12]. In the control arm, the cTTO was applied in the following way: After the respondents were introduced to the cTTO task for the health states BTD (‘conventional’ TTO) and the health states WTD (LT-TTO) using the wheelchair example and practised the cTTO task for three practise health states, the respondents valued ten health states beginning with the conventional TTO. The second iteration step in all cTTO tasks involved choosing between spending 0 years in full health in Life A and spending ten years in the EQ-5D-5L health state under valuation, followed by death (Life B). In other words, the respondents were asked whether they consider living in the health state for 10 years to be BTD or WTD (the sorting question). Using an iterative procedure, the respondents’ value of that health state was then identified by finding the number of years in full health (Life A) they considered equivalent to living for ten years in Life B (i.e. the cTTO values for the health states BTD range between 0 and 1). However, every time a respondent indicated to prefer immediate death over 10 years in the EQ-5D-5L health state, the EQ-VT changed to the WTD format (i.e. the LT-TTO). The LT-TTO approach applied in this valuation study involved a twenty year time frame: ten years of lead time followed by ten years in the EQ-5D-5L health state, followed by death (see Fig. 1). The respondents’ value of that health state was then investigated by finding the point of indifference (i.e. the cTTO values for health states WTD range between −1 and 0), again using iterative steps. The iteration scheme of the EQ-VT protocol is reported elsewhere [12].

In the test arm, the cTTO tasks for the health states BTD and the health states WTD were separated in the following way (*the BTD/WTD split*): After the respondents were solely introduced to the cTTO task for the health states BTD (‘conventional’ TTO) using the wheelchair example and practised the cTTO task for three practise states, the respondents valued ten health states beginning with the conventional TTO until the indifference point was identified. However, every time a respondent indicated to prefer immediate death over ten years in the EQ-5D-5L health state (in the second iteration step), that health state was temporarily parked. The interview continued with the BTD format (i.e. conventional TTO) for the remaining health states. After completing all BTD tasks, the WTD task (i.e. LT-TTO) was explained using the wheelchair example and afterwards all temporarily parked health states considered WTD were evaluated applying LT-TTO, again using iterative steps. However, the respondents still had the possibility to reconsider their initial classification of a health state being WTD and they were still able to switch to the BTD format.

In both study arms, the values derived by the cTTO range between 1 at maximum and −1 at minimum (see Fig. 1). The maximum cTTO value of 1 means that the respondent values the EQ-5D-5L health state to be as good as being in full health (i.e. no trading of life years). A value of 0 indicates that the respondent considers the EQ-5D-5L health state to be neither BTD nor WTD. All cTTO values below 0 imply that the respondent values the health state to be WTD. Thereby, the minimum value is restricted to −1 which means that the respondent can trade-off 10 years of full health at maximum (i.e. trading the whole lead time).

### Modification 3: the feedback module

**Fig. 2:**
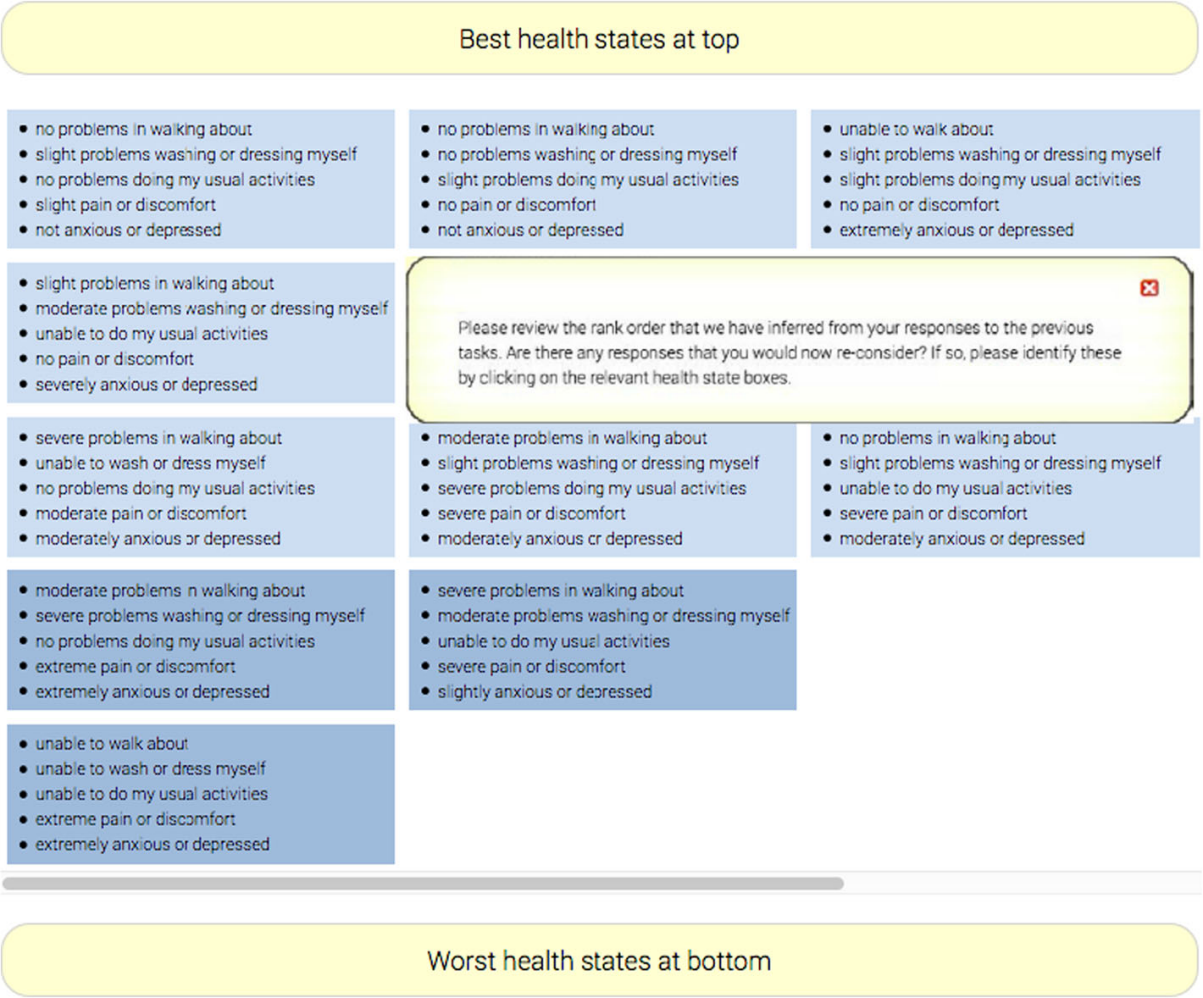
The feedback module

After completing the ten cTTO tasks, each respondent of the experimental test arm was presented with the rank ordering implied by their cTTO valuations in the *feedback module* (see Fig. 2). The respondents were asked to review their responses and to flag any that they felt should be reconsidered. However, those health states could only be flagged and not re-valued.

In the DCE tasks, respondents of both study arms were presented with a pair of EQ-5D-5L health states: A and B. They were asked to make a forced choice, expressing which of the two states was considered to be the better one. Results on DCE data are reported elsewhere, as this study focussed on the improvement of the cTTO task in the EQ-VT.

#### Study design

The general populations’ health state valuations were collected by a computer assisted personal interview (CAPI) survey in Germany. Interviews were conducted by five academic interviewers in the following two cities and surroundings located in different parts of Germany: Bielefeld and Hanover. Individuals were recruited through a mixed recruitment strategy, i.e. through personal contact, newspaper advertisement and from bulletins in the universities of Bielefeld and Hanover. Interviews were conducted in a public venue that was either a research office or a conference room.

The study sample consists of a control arm (*n* = 100; current EQ-VT version 1.1) and a test arm (*n* = 100; modified EQ-VT version 1.1). The control arm was asked to fill in the current version of the EQ-VT. The test arm was asked to use the modified version 1.1 of the EQ-VT (including the three modifications described above). The methodological procedure of the development of the German value set for the EQ-5D-5L will be based on the results of the tested modifications.

The data collection was organised in a two-step approach: (1) training and pilot phase and (2) field phase. In the first phase five academic interviewers were intensively trained in a daylong training in two groups. Each interviewer performed at least two pilot interviews for each version of the EQ-VT (n = 4) within one week after the interviewer training in order to test the methodological procedure (i.e. the technical implementation of the valuation task and the interviewer role). Afterwards the data were reviewed with the QC tool provided by the EuroQol Group to test the interviewers’ performance and to check for possible non-compliance to the interviewer instructions (e.g. the number of moves and the time spent explaining the cTTO task in the wheelchair example) [13, 21]. Following the interviewer instructions, each respondent should be exposed to a comparable survey experience (high protocol compliance). Differences in cTTO valuations should not be related to differences in the interview process but only to the differences between the respondents themselves. Careful and homogenous explanations of the wheelchair example with regard to the duration and the iterative steps are expected between interviewers and within an interviewer. A detailed description of the QC tool can be found elsewhere [13].

The interviewers got a written feedback for their pilot interviews. After discussing the interviewer performance with each interviewer, the interviewer entered the field phase. The participants were randomly assigned to one of the study arms by the EQ-VT. Therefore, each interviewer conducted interviews in the control arm as well as in the test arm in order to avoid an interviewer bias.

During the entire valuation study, the interviewers’ performance and the protocol compliance were evaluated using the QC tool on a regular basis. The results were regularly shared and discussed with each interviewer. Each interviewer had to perform at least 35 interviews to be included in the final interviewer team which aims at a harmonised learning effect of the five interviewers. Moreover, a written debriefing after the interview of every participant provided additional information on how the valuation interview and the performance of the interviewer were perceived by the respondents and ensured QC in terms of formative evaluation. The participants received the debriefing questionnaire including the initials of the respective interviewer and a stamped addresses envelope. The purpose of sending the questionnaire back by post was to avoid a bias due to the presence of the interviewer while answering the questionnaire and to get an honest appraisal of the interviewers. In addition to the regular communication with the interviewers during the data collection, a feedback round with all interviewers was held after the field phase on how the EQ-VT, the valuation tasks and the three modifications were perceived by the respondents and the interviewers in Germany.

#### Data analysis

Data analysis was performed to verify the hypotheses stated in Table 1 by gaining information on the quality of the collected cTTO data. Descriptive analyses (proportions for discrete variables, mean and standard deviation (SD) for continuous variables) were used to examine the following data: the background characteristics of the study sample, the time taken to complete the valuation interview and the single tasks (e.g. the wheelchair example, the cTTO tasks and the feedback module), and the distribution of the cTTO values. Differences between the study arms were identified via a *t*-test or a Mann-Whitney test (between study arm comparisons). Differences before and after the feedback module in the test arm were analysed using a *t*-test (within subject comparisons). The harmonisation level of the cTTO explanations within the study arms were analysed using means, SD and variation coefficients of the duration and the number of moves in the wheelchair example. The number of values at −1, −0.5, 0, 0.5 and 1 (spikes) was compared between the study arms as well as before and after the feedback module in the test arm using a proportion test (z-test applying Bonferroni correction).

To evaluate the data quality in terms of consistency of the cTTO responses, all pairs of the EQ-5D-5L health states were identified where one health state dominates the other one (i.e. health state A dominates health state B when health state A is better than health state B on at least one dimension, and not worse than health state B on any of the remaining dimensions). An inconsistency was defined as an observation of B (i.e. the dominated health state) being given a higher value by a respondent than A (i.e. the dominant health state). The level of inconsistencies was compared using a Chi-squared test for homogeneity between the study arms. The McNemar test was used for analysing the effect of the feedback module on the level of inconsistencies.

Statistical analyses were performed in STATA. The free text answers of the debriefing questionnaire were analysed based on a qualitative content analysis.

## Results

A total of 200 interviews (*n* = 100 in each study arm) were performed from 18^th^ June – 11^th^ August 2014. Each interviewer was randomly allocated to both study arms in almost equal parts (the ratio of control arm to test arm ranged from 46:54 to 53:47). Mean interview time of the valuation interview and the country-specific questionnaire including further background questions specific to the German valuation study was 59.09 minutes, whereby the test arm was about 13 min longer (control arm: 52.58 min, test arm: 65.6 min).

### Characteristics of the sample

Table 2 gives an overview of the demographics of the study sample. Mean age of the respondents was 37.89 years. The majority of the respondents are aged under 40 years in both study arms. Statistically significant differences in terms of age and gender were observed between the study arms. Both sexes were included in the study, but women were slightly overrepresented in general (58%) and in the test arm (68%). In the control arm the contrary can be noticed (48% female). Study participants were mostly higher educated in both arms (83%). Study participants were in almost equal parts employed or non-employed.

**Table 2:** Study sample

		Control arm (*n* = 100)	Test arm (*n* = 100)	Total (*n* = 200)	Sig.
Age, *n* (%)	Mean (SD)	38.32 (16.34)	37.46 (15.41)	37.89 (15.88)	*
	18–24	13 (13%)	18 (18%)	31 (15.5%)	
	25–29	33 (33%)	34 (34%)	67 (33.5%)	
	30–39	18 (18%)	13 (13%)	31 (15.5%)	
	40–49	10 (10%)	7 (7%)	17 (8.5%)	
	50–64	17 (17%)	25 (25%)	42 (21%)	
	65–74	6 (6%)	1 (1%)	7 (3.5%)	
	> = 75	3 (3%)	2 (2%)	5 (2.5%)	
Gender, *n* (%)	Female	48 (48%)	68 (68%)	116 (58%)	*
	Male	52 (52%)	32 (32%)	84 (42%)	
Education, *n* (%)	Lower education^a^	6 (6%)	7 (7%)	13 (6.5%)	ns.
	Middle education^b^	11 (11%)	10 (10%)	21 (10.5%)	
	Higher education^c^	83 (83%)	83 (83%)	166 (83%)	
Employment status, *n* (%)	Employed	53 (53%)	52 (52%)	105 (52.5%)	ns.
	Non-employed	47 (47%)	48 (48%)	95 (47.5%)	

^a^lower education: with or without secondary general school certificate, ^b^middle education: intermediate school certificate, ^c^higher education: entrance qualification for universities of applied sciences, university entrance qualification, *p < 0.05﻿

Self-reported health using the EQ-5D-5L showed that about half of the sample reported no problems in any dimension (11111): 52% in the control arm and 49% in the test arm. Frequencies of reported problems varied from 2% in the dimension self-care to 41.5% in the dimension pain/discomfort (see Table 3). Small but statistically significant differences were observed between the study arms. However, there is no clear pattern of significantly higher frequency of reported problems in the five dimensions of the descriptive system of the EQ-5D-5L in one of the study arms. Mean self-reported health using the EQ VAS was 87.87. Self-reported mean VAS was lower in the control arm than in the test arm (86.74 vs. 89.00; *p* ≤ 0.001).

**Table 3:** Self-reported health using EQ-5D-5L

		Control arm (*n* = 100)	Test arm (*n* = 100)	Total (*n* = 200)	Sig.
Mobility, *n* (%)	No problems	81 (81%)	87 (87%)	168 (84%)	*
	Slight problems	16 (16%)	9 (9%)	25 (12.5%)	
	Moderate problems	2 (2%)	4 (4%)	6 (3%)	
	Severe problems	1 (1%)	0 (0%)	1 (0.5%)	
	Unable	0 (0%)	0 (0%)	0 (0%)	
Self-care, *n* (%)	No problems	99 (99%)	97 (97%)	196 (98%)	ns.
	Slight problems	1 (1%)	2 (2%)	3 (1.5%)	
	Moderate problems	0 (0%)	1 (1%)	1 (0.5%)	
	Severe problems	0 (0%)	0 (0%)	0 (0%)	
	Unable	0 (0%)	0 (0%)	0 (0%)	
Usual activities, *n* (%)	No problems	79 (79%)	89 (89%)	168 (84%)	*
	Slight problems	20 (20%)	7 (7%)	27 (13.5%)	
	Moderate problems	1 (1%)	4 (4%)	5 (2.5%)	
	Severe problems	0 (0%)	0 (0%)	0 (0%)	
	Unable	0 (0%)	0 (0%)	0 (0%)	
Pain/discomfort, *n* (%)	No problems	55 (55%)	62 (62%)	117 (58.5%)	ns.
	Slight problems	33 (33%)	31 (31%)	64 (32%)	
	Moderate problems	11 (11%)	6 (6%)	17 (8.5%)	
	Severe problems	1 (1%)	1 (1%)	2 (1%)	
	Extreme problems	0 (0%)	0 (0%)	0 (0%)	
Anxiety/depression, *n* (%)	No problems	88 (88%)	74 (74%)	162 (81%)	*
	Slight problems	10 (10%)	21 (21%)	31 (15.5%)	
	Moderate problems	2 (2%)	4 (4%)	6 (3%)	
	Severe problems	0 (0%)	1 (1%)	1 (0.5%)	
	Extreme problems	0 (0%)	0 (0%)	0 (0%)	
EQ VAS	Mean (SD)	86.74 (10.05)	89.00 (9.19)	87.87 (9.69)	*

### Wheelchair example

All interviewers performed interviews with both software versions: EQ-VT version 1.1 (control arm) and modified EQ-VT version 1.1 (test arm). Table 4 provides an overview of the duration and the number of moves in the wheelchair example and homogeneity of wheelchair example within both study arms. The duration of the wheelchair example was lower in the test arm (447 s) compared to the control arm (477 s). The number of iteration steps (moves) to explain the cTTO tasks in the wheelchair example showed a similar pattern. The variation coefficients were lower in the test arm regarding duration, i.e. there was a higher homogeneity of duration of the cTTO explanation in the test arm. No statistically significant differences were observed between the study arms for the mean number of moves and the duration of the wheelchair example, except for the duration of the WTD element (138 s in the control arm vs. 100 s in the test arm).

**Table 4:** Duration and number moves in the wheelchair example

		Control arm (*n* = 100)		Test arm (*n* = 100)		Total (*n* = 200)		Sig.
		Mean (SD)	Variation Coeff.	Mean (SD)	Variation Coeff.	Mean (SD)	Variation Coeff.	
Duration (sec.)	Total time	477.65 (260.19)	0.544	447.30 (182.20)	0.407	462.47 (224.55)	0.486	ns.
	BTD time	338.80 (196.62)	0.580	346.87 (147.11)	0.424	342.84 (173.25)	0.505	ns.
	WTD time	138.85 (114.38)	0.824	100.43 (74.78)	0.745	119.64 (98.29)	0.822	*
Number of moves	Total moves	47.81 (23.63)	0.494	43.48 (24.78)	0.570	45.64 (24.24)	0.531	ns.
	BTD moves	29.90 (15.26)	0.510	28.43 (15.28)	0.538	29.17 (15.25)	0.523	ns.
	WTD moves	17.91 (12.46)	0.696	15.05 (13.04)	0.866	16.48 (12.80)	0.777	ns.

#### cTTO data

The mean duration of time spent on valuing an EQ-5D-5L health state in the cTTO tasks was 1.85 minutes, whereby the respondents of the test arm spent slightly more time for each cTTO task (1.91 min in the test arm vs. 1.71 min in the control arm; p ≤ 0.001). On average respondents had 7.1 moves before they reached their point of indifference in the cTTO tasks (control arm: 6.9; test arm: 7.3; p ≤ 0.05). In the test arm, completing of the feedback module took an average of 3.37 min. In the feedback module, 40 out of 100 respondents (40%) of the test arm removed one or more responses. After valuing ten health states a total of 6.7% of the responses (67 health states) were removed by the respondents from the rank ordering that they considered ‘wrong’. Most frequently the values for 55555 were removed from the rank ordering.

**Fig. 3:**
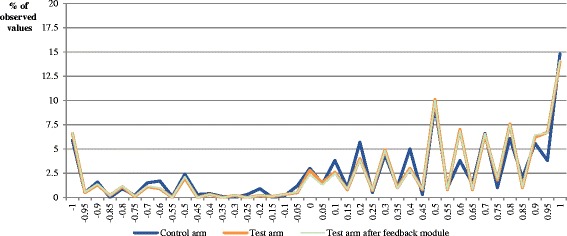
cTTO value distribution

Figure 3 gives an overview of the observed cTTO value distribution. There are similar patterns for the cTTO value distribution in both study arms. The majority of the cTTO values are positive, whereas 16.8% of the health states are valued to be WTD (control arm: 18.2%, test arm: 15.4%; *p* > 0.05). The number of respondents who provided at least one WTD response is significantly higher in the test arm (71%) than in the control arm (62%; *p* ≤ 0.001). There is no statistically significant difference in the number of WTD values in total and per person before and after the feedback module (*p* > 0.05). Differences in the percentage of values at −1, −0.5, 0, 0.5 and 1 between the study arms are small and not statistically significant before and after a Bonferroni correction (*p* > 0.05). There is almost no clustering of valuations at certain values on the scale (−1, −0.5, 0, 0.5 and 1). The highest proportion of values can be observed at 1 (control arm: 14.8%, test arm: 14%). The proportion of health states valued at 0 is about 3% in the control and the test arm (*p* > 0.05). After the feedback module, the proportion of health states assigned at 0 is slightly reduced (2.36%). However, the differences in percentage of values at −1, −0.5, 0, 0.5 and 1 before and after the feedback module are not statistically significant before and after a Bonferroni correction (*p* > 0.05).

**Fig. 4:**
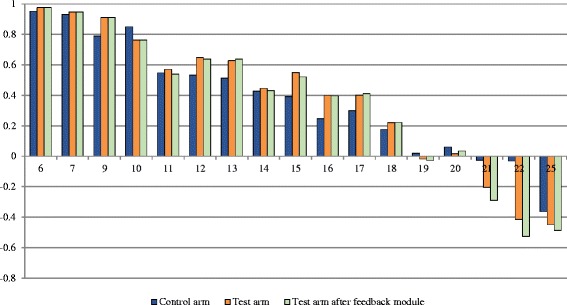
Mean cTTO value by severity

As shown in Fig. 4, the higher the severity level (i.e. the sum of levels of all dimensions) the lower the mean cTTO value in the control arm and the test arm (before and after the feedback module). No statistically significant differences were found between the study arms (except for severity 15, *p* < 0.05). There is a growing variance with increasing severity, i.e. the respondents’ opinions on severe health states differ more than for mild health states. The cTTO mean score in the control arm varies from 0.95 for the mildest health states with severity of 6 over 0.51 for health states with medium severity level 13 to −0.36 for health states with severity 25 (i.e. the health state 55555). The range of mean cTTO values is marginally higher in the test arm (−0.44 for severity 25 to 0.97 for severity 6). There is a higher discrimination for severe health states in the test arm (see Fig. 4). The feedback module had no significant effect on the mean cTTO values (*p* > 0.05).

**Fig. 5:**
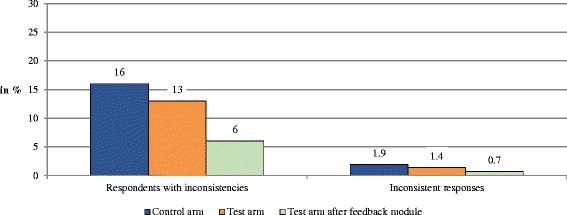
Inconsistencies of cTTO values

Figure 5 compares the percentage of respondents with at least one inconsistency and the percentage of inconsistent cTTO responses. In the control arm, 16% of respondents had at least one inconsistency in their cTTO responses. The proportion of respondents with at least one inconsistent response was lower in the test arm (13%; *p* > 0.05) and significantly reduced after the feedback module (6%; *p* ≤ 0.001). In total, 1.9% (control arm) and 1.4% (test arm) of all cTTO responses were inconsistent (*p* > 0.05). The level of inconsistencies was significantly reduced by 50% after the feedback module (before the feedback module: 1.4%, after the feedback module: 0.7%; *p* = 0.01). Moreover, the proportion of respondents who did not value the health state 55555 the lowest is 6% in both study arms. After the feedback module, 1.08% of the respondents still did not value health state 55555 the lowest (*p* < 0.05).

#### Debriefing of the respondents and the interviewers

An additional written debriefing of the respondents provided information on the performance of the five interviewers perceived by the respondents (*n* = 96; 48% response rate). According to the results of the debriefing, 92.7% of the interviewers fully explained the background and the aim of this valuation study. In total, 64.58% of the respondents strongly agreed that the duration of the interview was appropriate. However, the content analysis of the open questions showed that many respondents of the test arm evaluated the ranking task of the ten health states to last too long and to be too difficult to decide on at the beginning of the interview: *“I was overburdened when I was ask to rank 10 health states and the ‘death’ at the beginning of the interview because the health states looked so similar. It was really difficult to decide on the ranking based on my preferences as I never thought about those health states before”*. The majority of the respondents described the cTTO tasks to be clear and comprehensible (strongly agree: 50%, rather agree: 43.75%). The respondents of the test arm mentioned in the comment box that the possibility to park severe health states facilitates the valuation of these health states at the end of the cTTO tasks. Moreover, the majority of the respondents of the test arm liked the possibility to review their cTTO responses in the feedback module and to correct for wrong answers given (strongly agree: 60%, rather agree: 26%). “*During the [cTTO] tasks I could not remember all of my previous answers. It was good that I could see the ranking based on my answers at the end of the section and that I could make corrections*”.

Besides the regular communication during the whole study, a feedback group discussion was held with all interviewers. The interviewers themselves enjoyed performing the interviews and got positive feedback during and after the interviews. Both software versions used, the EQ-VT version 1.1 and the modified EQ-VT version 1.1, in combination with the interviewer instructions and the feedback from the routine QC checks were regarded as feasible and comprehensible. Evaluating the different software versions, the interviewers criticised the high cognitive burden of the ranking task at the early stage of the interviews. Many respondents were overstrained and needed a lot of support to fulfil the ranking task. Besides the increased complexity and duration of the whole valuation interview, the interviewers felt that the ranking task did not facilitate the following cTTO tasks and judged this modification as “*unnecessary prolongation of the interview*”. Regarding the modification of the BTD/WTD split, the interviewers reported that (the split of) the wheelchair example in the test arm was regarded as being more feasible. It was easier for them to firstly explain the BTD task and later on the WTD task as there was no overburdening of the respondents at the wheelchair example. Explaining conventional TTO for BTD and LT-TTO for WTD simultaneously was regarded as an interruption of thinking in the current EQ-VT version 1.1 (used in the control arm of this study). “*Moreover, many respondents in the test arm liked to temporally park severe health states, to continue with mild health states [in the BTD format] and to value the severe health states at the end again [in the WTD format]. These decisions were judged as being easier”.* The feedback module was regarded as a useful modification, especially for the respondents who would like to correct their answers as this is not possible in the current EQ-VT 1.1. However, other respondents must be strongly encouraged by the interviewers to review the ten health states again.

## Discussion

This paper presents the results of the collected cTTO data of an exploratory German EQ-5D-5L valuation study. The improved valuation protocol combined with an intensive interviewer training and a close data monitoring showed a high feasibility and acceptability by the respondents of the general population as well as the interviewers in Germany. Cognitive debriefing exercises provided additional information on how the EQ-VT version 1.1, the valuation tasks and the three modifications were perceived by the respondents and the interviewers.

The different steps of strict QC contributed to a high data quality in both study arms. Compared to the first wave of EQ-5D-5L valuation studies using the EQ-VT version 1.0 [9, 11] there were fewer data issues in both study arms:

- the presented data showed few inconsistencies in total as well as those concerning the worst health state 55555 (at least 20% in the first valuation studies versus 6% in this study),
- a few “spikes” at 0.5 and (−)1 (in the first valuation studies spikes at −1, −0.5, 0, 0.5 and 1),
- a small gap between the mildest health state and full health (e.g. 0.90-0.92 for severity 6 in the valuation study in the Netherlands versus 0.96 in this study),
- more WTD responses (16.8% in this study) [9–11, 22].

As this clear improvement of data quality can be identified in both study arms, it might be related to the three modifications which were added to the protocol and led to the EQ-VT version 1.1 (i.e. routine QC checks, adding three practise health states and a prompt after each answer). However, the gap between −0.5 and 0 still exists. This might be partly related to the iterative routing of the EQ-VT: each time the respondent moves to the WTD element, the second iteration step is 0.5 and the next steps are half-year steps in contrast to annual steps in the BTD element, i.e. trading between 5 and 0 years of the lead time requires more iterative steps compared to the BTD element. Alternative routing procedures of the WTD element which is adapted to the BTD element should be an objective of future research around the EQ-VT.

It has to be restrictively mentioned that the modification of the three add-ons was tested simultaneously in this study. Therefore the results of other experimental studies of the EQ-VT research methodology programme testing a single modification each were additionally included in the final decision of the methodology for the German EQ-5D-5L valuation study. In those studies each experimental arm applied only one modification to the EQ-VT [9].

Firstly, it was hypothesised that the introduction of the *ranking task* prior to the cTTO valuations will reduce the inconsistencies and improves the overall data quality. The test arm resulted in a higher data quality in terms of fewer inconsistencies. However, the hypothesis cannot be verified based on the quantitative data analysis of this study as the EQ-VT version of the test arm also included two further modifications. The debriefing of the respondents and the interviewers showed that the ranking task itself implied a high cognitive burden to the respondents and increased the complexity as well as the duration of the interview (about 10 min as recorded in the EQ-VT). On average, the ranking task prolonged the interviews about 8 min in other countries and increased the complexity of the studies [9]. The EQ-VT methodology research programme of the EuroQol Group came to the result that the ranking task with sorting reduced the face validity of the data. The feasibility and acceptability to respondents and interviewers are low [9, 21]. Overall, this led to the decision not to implement the ranking task in the main valuation study in Germany.

Secondly, *separating the BTD and WTD TTO tasks* tested the hypothesis that this will reduce the inconsistencies of the valuations and improves the overall data quality. The test arm resulted in a higher data quality in terms of higher values for mild heath states (0.97 versus 0.95 for severity level 6) and fewer inconsistencies (1.4% versus 1.9%). The reduction of spikes compared to the first wave of valuation studies can be noticed in both study arms. The discriminative capability for severe health states is higher in the test arm in comparison to the control arm. The total amount of WTD values in the test arm was increased (15.4%) compared to the first wave of valuation studies but it was slightly lower than in the control arm (18.2%). However, the tested hypothesis also cannot be verified solely on the quantitative data analysis of this study as the test arm applied a software including further modifications. The BTD/WTD split study of the EQ-VT methodology research programme in the Netherlands found that it promotes consistency in the data [9, 22]. As the results of an equivalent study in Hong Kong did not confirm this, no clear recommendation could be derived from the EQ-VT methodology research programme. However, the debriefing of the respondents in this study showed that they were in favour of temporally parking severe health states which made it easier for them to decide on these health states after valuing all BTD health states. Moreover, the interviewers reported that it was easier for them to split the explanation of the BTD element and the WTD element as there was no overburdening of the respondents during the wheelchair example. They felt that explaining both cTTO tasks at the same time, i.e. the conventional TTO for BTD and the LT-TTO for WTD, is an interruption of thinking in the current EQ-VT version 1.1. In accordance with the debriefing results of the interviewers, the data analysis confirmed a significantly lower and more homogenous duration of the WTD element in the wheelchair example while keeping an adequate level of the protocol compliance. In summary, based on the results of this exploratory study in Germany and the study in the Netherlands it was decided to implement the BTD/WTD split in the main valuation study in Germany.

Thirdly, it was tested whether the addition of the *feedback module*, i.e. presenting respondents with the rank ordering implied by their cTTO valuations, will reduce inconsistencies as they will identify and flag problematic valuations for removal from the data. The feedback module increases interview length by 3.37 min. The feedback module had no significant effect on mean cTTO values, but significantly reduced the level of inconsistencies from 1.4% (test arm before feedback module) to 0.7%. So, the stated hypothesis can be verified based on the presented data. Other studies, like in Spain and the Netherlands, also confirm that this modification statistically increased the proportion of consistent respondents [9, 22]. Moreover, respondents of this study reported that they liked to remove problematic valuations after having seen all ten health states. So, the feedback module will be implemented in the main EQ-5D-5L valuation study in Germany.

Besides the above stated limitation of testing three modifications at the same time, there are further limitations to be mentioned. Respondents were partly recruited using convenience sampling within the university context. Compared to the general population in Germany, young and highly educated persons were therefore overrepresented in this study [23]. Thus it would be unjustified to suggest that the data are representative of the whole German population. The feasibility and acceptability of the improved EQ-5D-5L valuation protocol need to be further investigated in future valuation studies, especially in older and lower educated people. However, for the purpose of this experimental study a strict representativeness of the German general population was not required. Moreover, even though the participants were randomly assigned to one of the study arms by the EQ-VT, there are differences between the study arms regarding socio-demographics and health status which might account for some of the observed variation in respondents’ values. However, the interviewers conducted interviews in the control arm as well as in the test arm to avoid further differences between the study arms due to an interviewer bias.

According to the EQ-5D-5L valuation protocol 1.1, the LT-TTO used in this study applied a twenty year time frame (i.e. ten years of lead time followed by ten years in impaired health), which implied that it was possible to trade-off a maximum of ten years of lead time (minimum value of −1). However, there were no information collected whether the value −1 or even a lower value was the respondents’ preference. Therefore, it cannot be excluded that respondents would have trade-off even more life years in full health than the maximum possible (longer lead time), in which case their value would be lower than −1 [8, 24]. Methods have been recently developed to model value sets taking into account those cTTO data bounded at −1 (censoring) [8, 10, 24]. However, additional (trade-off) questions could be asked of those respondents who traded the whole lead time as object of research in future EQ-5D-5L valuation studies.

The influence of time discounting on cTTO values was not regarded in this experimental study as the focus was on testing the EQ-VT 1.1 and the further three modifications. Thereby, a possibly resulting downward bias cannot be excluded [15, 25]. More exploratory research is required to develop ways of controlling for time preferences in the estimation of cTTO tariffs. Thereby different discount rate elicitation procedures should be developed and their feasibility as well as their validity in general population samples should be explored [15, 16, 25].

## Conclusions

This exploratory study showed the feasibility and acceptability of the improved EQ-5D-5L valuation protocol in Germany and resulted in higher data quality in both study arms compared to the first wave of EQ-5D-5L valuation studies. It can be concluded that the modifications of the international EQ-5D-5L valuation protocol from EQ-VT version 1.0 to EQ-VT version 1.1 had a positive impact on the data quality and provided evidence for continuing with the cTTO-based valuation protocol in future valuation studies.

Based on the results of this study and other studies of the EQ-VT research methodology programme [9], it was decided to implement two modifications in the main EQ-5D-5L valuation study in Germany: splitting the TTO tasks for the health states BTD and WTD as well as the inclusion of the feedback module. The BTD / WTD split version showed a higher feasibility and acceptance by the participants as well as the interviewers and the feedback module resulted in the lowering of inconsistencies.

This study assured the feasibility and acceptability of the improved EQ-5D-5L valuation protocol in Germany and builds an empirical basis for the coming valuation study in Germany. The German value set for the EQ-5D-5L will be expected in 2017. The final German value set might facilitate the use of the EQ-5D-5L in a range of applications for health care policy and clinical assessment in Germany and other German-speaking countries.

## Abbreviations

BTD: Better than dead
CAPI: Computer assisted personal interview
cTTO: Composite time trade-off
DCE: Discrete choice experiment
EQ-VT: EuroQol – Valuation Technology
HrQoL: Health-related quality of life
LT-TTO: Lead time – time trade-off
ns: Non-significant
SD: Standard deviation
TTO: Time trade-off
VAS: Visual analogue scale
WTD: Worse than dead
